# Metabolomic Characteristics of Liver and Cecum Contents in High-Fat-Diet-Induced Obese Mice Intervened with *Lactobacillus plantarum* FRT10

**DOI:** 10.3390/foods11162491

**Published:** 2022-08-18

**Authors:** Hongying Cai, Daojie Li, Liye Song, Xin Xu, Yunsheng Han, Kun Meng, Zhiguo Wen, Peilong Yang

**Affiliations:** 1 Key Laboratory of Feed Biotechnology of Ministry of Agriculture and Rural Affairs, Institute of Feed Research, Chinese Academy of Agricultural Sciences, Beijing 100081, China; 2 National Engineering Research Center of Biological Feed, Beijing 100081, China

**Keywords:** obesity, *Lactobacillus plantarum* FRT10, UHPLC-QTOF/MS, metabolomics, biomarker, gut–liver axis

## Abstract

Obesity has become a major social problem related to health and quality of life. Our previous work demonstrated that *Lactobacillus plantarum* FRT10 alleviated obesity in high-fat diet (HFD)-fed mice by alleviating gut dysbiosis. However, the underlying functions of FRT10 in regulating liver and cecum contents metabolism remain unknown. Liver and cecum contents metabonomics combined with pathway analysis based on ultraperformance liquid chromatography-quadrupole-time-of-flight mass spectrometry (UHPLC-Q-TOF/MS) were performed to evaluate the alterations of metabolic profiles between obese control mice and obese mice in FRT10-treated groups. The orthogonal partial least squares discriminant analysis (OPLS-DA) score plots showed that there were significant differences in cecum contents and liver markers between experimental groups. In total, 26 potential biomarkers were identified in the liver and 15 in cecum contents that could explain the effect of FRT10 addition in HFD-fed mice. In addition, gut–liver axis analysis indicated that there was a strong correlation between cecum contents metabolites and hepatic metabolites. The mechanism of FRT10 against obesity might be related to the alterations in glycerophospholipid metabolism, primary bile acid biosynthesis, amino metabolism, and purine and pyrimidine metabolism. Studies on these metabolites could help us better understand the role of FRT10 in obesity induced by HFD.

## 1. Introduction

Obesity caused by diet is considered a serious health problem that increases the risk of many chronic diseases such as cardiovascular diseases, type 2 diabetes, hypertension, and mental disorders [1]. Obesity is characterized by abnormal or excessive accumulation of fat as a result of a chronic imbalance between energy intake and expenditure [2]. Increasing reports indicate that gut microbiota plays an important role in metabolic disorders associated with obesity and liver diseases [3,4]. It is well known that gut microbiota can improve the host’s energy production from food digestion, alter choline metabolism, and modulate the enterohepatic circulation of bile acids [5]. Similarly, change in the microbiota structure are known to destroy the integrity of the gut barrier, allowing subsequent bacteria metabolites and their components, along with nutrients and other signals, to be transported to the liver through the portal circulation and change gut–liver crosstalk, thereby inducing downstream inflammatory pathways involved in the progression of obesity. The liver plays a key role in defending against substances from the gut, which is defined as the gut–liver axis [6,7]. Recently, the gut microbiota has attracted much attention as a therapeutic approach to combat obesity. The complicated relationship between gut microbiota and obesity opens up an attractive window for finding successful and safe treatments for obesity.

*Lactobacillus* is a significant part of the gut microbiota, which can produce metabolites to inhibit other microorganisms’ growth in the intestinal mucosa, strengthen the integrity of the gut barrier, and regulate mucosal homeostasis [8,9]. In addition, *Lactobacillus* co-exists with intestinal stem cells to regulate epithelium cell regeneration [8,10], which is essential for maintaining the gut barrier and gut homeostasis. *Lactobacillus plantarum* is a kind of multifunctional lactic acid bacterium, which carries out many biological activities, including alleviating symptoms of irritable bowel syndrome [11], reducing total cholesterol [12], and improving immune function [13]. The treatment of obesity with *L. plantarum* has been proved to be an efficacious agent for preventing the worsening of obesity [14,15,16]. Although the beneficial effects of *L. plantarum* have been well documented, the detailed mechanisms by which *L. plantarum* combat obesity are not fully understood to date. Metabolomics is a comprehensive quantitative and qualitative analysis of metabolite alterations in cells, tissues, or biological fluids to discover potential biomarkers. The approach serves as the endpoint of the ‘-omics’ cascade after genomics, transcriptomics, and proteomics and plays an increasingly important role in the high-throughput studies of global metabolites that could be used to diagnose and monitor disease progression [17,18]. Previous studies of obesity have intervened with *Lactobacillus* species and identified a number of biomarkers [14,19,20], but none have monitored *L. plantarum* supplementation to produce as many metabolic alterations in a model of obesity. Therefore, understanding the role of *L. plantarum* in obesity metabolism from a few metabolites is limited.

In our previous study, FRT10 intervention significantly reduced body weight gain, liver weight, serum TG, hepatic TG, and ALT in high-fat-diet-induced obese mice [11]. FRT10 intervention alleviated obesity by modulating the PPARα signal pathway and reshaping gut microbiota composition [11]. In addition, we found that a high-fat diet (HFD) induced significant metabolomic changes in liver tissue and cecum contents in obese mice [21]. In the present study, a comprehensive analysis was conducted to investigate the possible characteristic metabolites altered by FRT10 through cecum contents and liver metabolomics in obese mice. We aimed to better understand the complex correlation between the gut and liver and how it modulates systemic metabolic alterations in HFD-induced obesity. Results from the research will provide a scientific basis for *L. plantarum* FRT10 application in the treatment of obesity.

## 2. Materials and Methods

### 2.1. L. Plantarum FRT10 Culture

*L. plantarum* FRT10 was isolated and identified from Poland sour dough and deposited in the Chinese General Microorganism Collection Center (accession 17956; CGMCC, Beijing, China). The strain was grown in MRS broth at 37 °C for 24 h, centrifuged at 4 °C, 5000× *g*, 10 min, and the cells were collected and washed with 0.9% saline buffer three times. Then, the strain was adjusted to 1 × 10^9^ and 1 × 10^10^ CFU/mL in saline buffer for gavage, respectively.

### 2.2. Animals and Experiment Design

Twenty-seven 7-week-old, specific pathogen-free (SPF) female Kunming mice were obtained from Vital River Laboratory Animal Technology Co., Ltd. (Beijing, China). After one week of adjustment, as previously reported [22], the mice were fed with HFD consisting of 67% (*w*/*w*) normal diet, 20% sucrose, 10% lard, 2.5% cholesterol, and 0.5% sodium cholate with minor modifications. After 8 weeks of dietary intervention, HFD mice were randomly divided into 3 subgroups (n = 9 in each group) according to their body weight with no significant difference and continued to be fed HFD: (1) HF group: high-fat diet group, with gavage daily 0.9% saline as the obese control; (2) HF10L group: HFD plus daily administration of 0.2 mL of 1 × 10^9^ CFU/mL *L. plantarum* FRT10; and (3) HF10H group: HFD plus daily administration of 0.2 mL of 1 × 10^10^ CFU/mL *L. plantarum* FRT10. The gavage experiment lasted for 8 weeks. The animal study was reviewed and approved by the Laboratory Animal Ethical Committee and its inspection by the Feed Research Institute, Chinese Academy of Agricultural Sciences (Approval No. AEC-CAAS-20090609).

### 2.3. Collection of Liver Tissue and Cecum Contents Samples

On the last day of the experiment, mice were sacrificed after 6 h of fasting. Wash liver tissues with ice-cold buffered saline. Then, the liver tissues and cecum contents were collected, weighted, frozen in liquid nitrogen, and stored at −80 °C for further analysis.

### 2.4. Hepatic Metabolomics Analysis

The frozen liver tissues (60 mg) were thawed at 4 °C; 800 μL of precooled methanol/acetonitrile (1:1, *v*/*v*) was added to extract metabolites and then homogenized. Ultrasonic crushing was performed 2 times on ice for 30 min and placed at −20 °C for 1 h, and then centrifuged at 4 °C, 14,000× *g* for 20 min. The supernatant was freeze-dried and kept at −80 °C until further research.

Untargeted metabolic profiling analysis was performed using ultraperformance liquid chromatography quadrupole time-of-flight tandem mass spectrometry (UHPLC-Q-TOF/MS). Agilent 1290 Infinity LC ultra-high pressure lipid chromatography (UHPLC) system (Agilent, Santa-Clara, CA, USA) and AB SCIEX Triple TOF 6600 System (AB SCIEX, Framingham, MA, USA) were used for LC-MS analysis. LC conditions were set as follows: ACQUITY UPLC HSS T3 column (2.1 × 100 nm, 1.8 μm, Waters MS Technologies, Manchester, UK). The column was maintained at 25 °C, and separation was achieved using the following gradient: 95% B over 0–1.0 min, 95–65% B over 1.0–14.0 min, 65–40% B over 14.0–16.0 min, 40% B over 16.0–18.0 min, 40–95% B over 19.0–18.1 min, 95% B over 18.1–23.0 min. The flow rate was 300 μL/min. A (0.25 mM ammonium acetate and 25 mM ammonium hydroxide in water) and B (acetonitrile) were used as mobile phase. The automatic sampler was maintained at 4 °C throughout the analysis.

The mass spectrometric data were collected using a Triple TOFTM 5600 system (AB/Sciex, Foster City, CA, USA) equipped with an electrospray ionization (ESI) source operating in either the positive (ESI+) and negative (ESI−) ion mode. The ESI conditions after separation by UHPLC chromatographic were as follows: 60 psi for ion source gas 1 (Gas 1), 60 psi for ion source gas 2 (Gas 2), 30 psi for curtain gas (Cur), 600 °C for source temperature, and ±5500 V for ion sapary voltage floating (ISVF). TOF/MS scan *m*/*z* range is from 60 Da to 1000 Da, product ion scan *m*/*z* range is from 25 Da to 1000 Da. TOF MS scan accumulation time is 0.20 s/spectra, product ion scan accumulation time is 0.05 s/spectra. Information-dependent acquisition (IDA) with the high-sensitivity modes was used for MS/MS data recording. The IDA parameters were as follows: exclude isotopes within 4 Da, the number of candidate ions monitored per cycle: 6. Monitoring of the system stability and data quality was performed by quality control (QC) samples.

### 2.5. Cecum Contents Metabolomics Analysis

Cecal contents were freeze-dried and then crushed. Then, 1000 μL of the extract solution (acetonitrile–methanol–water = 2:2:1) and 1 g/mL internal standard was placed in 50 mg cecal contents in an EP tube. Homogenate and ultrasonic extraction were performed after vortex-mixing for 30 s and centrifuged at 4 °C, 12,000× *g* for 15 min. The 0.22 μm nylon filter was used to filter the supernatant, and 2 μL was injected for analysis. The QC samples were prepared by mixing the supernatants of all samples in equal quantities.

The UHPLC system (1290, Agilent Technologies) combined with a UPLC HSS T3 column (2.1 mm × 100 mm, 1.8 μm) and Q Exactive mass spectrometer (Thermo Fisher, Waltham, MA, USA) was used for chromatographic separation. The flow rate was 500 μL/min, using a mobile phase of solvent A (0.1% (*v*/*v*) formic acid) for ESI+ mode, 5 mmol/L ammonium acetate for ESI− mode, and acetonitrile was used for the mobile phase B. The elution gradient was set as follows: 1% B over 0–1.0 min, 1–99% B over 1.0–8.0 min, 99% B over 8.0–10.0 min, 99~1% B over 10.0–10.1 min, 1% B over 10.1–12 min. The MS data were collected from *m*/*z* 50–1200 Da with ESI source, including ESI+ and ESI− modes. Xcalibur 4.0.27 software (Thermo Fisher, Waltham, MA, USA) based on the IDA function was used for MS spectra acquisition. The ionization source conditions were as follows: 45 Arb for sheath gas flow rate, 15Arb for Aux gas flow rate, 400 °C for capillary temperature, 70,000 for full MS resolution, 17,500 for MS/MS resolution, 20/40/60 in NCE mode for collision energy, positive mode 4000 V or negative mode −3600 V for spray Voltage.

### 2.6. Data Processing and Statistical Analysis

Baseline information analyses were performed as described in our previous report [21]. The original data of UHPLC-Q-TOF/MS were converted to the mzXML format via ProteoWizard before importing the data into the XCMS program for peak detection, extraction, alignment, and integration. The metabolites were then annotated using an in-house MS2 database (BiotreeDB).To identify global differences in metabolic profiles between groups, supervised orthogonal partial least squares discriminant analysis (OPLS-DA) was employed. The robustness of the OPLS-DA model was evaluated by 7-fold cross-validation and response 200 permutation test. By multidimensional analyses of OPLS-DA and Student *t*-test, the identified metabolites were selected as differentiated metabolites, which all met the variable importance for the projection (VIP) >1 and *p* value < 0.05. Then, metabolites with significant differences (*p* < 0.05) were analyzed in the Kyoto Encyclopedia of Genes and Genomes (KEGG, http://www.kegg.jp (accessed on 29 October 2019)) databases. In addition, the metabolic pathways of important identified metabolites were queried against the online KEGG database. Only functional categories and pathways with *p* < 0.05 were considered significantly enriched.

## 3. Results

### 3.1. Multivariate Statistical Analysis of Liver Metabolites

**Figure 1:**
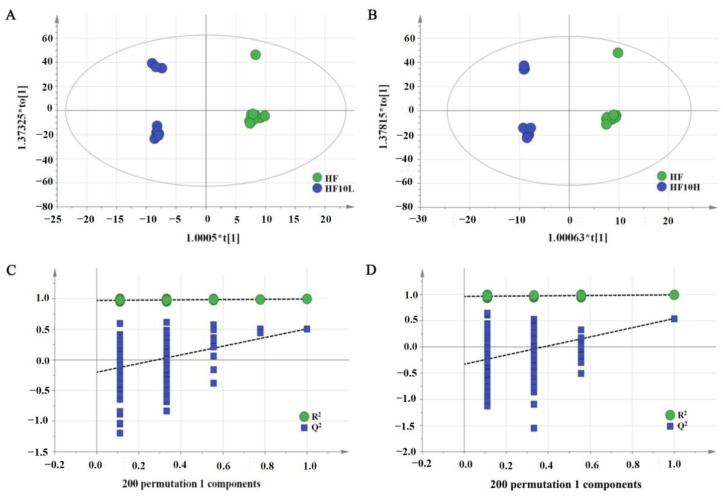
Score plots and permutation test of liver metabolites after *L. plantarum* FRT10 intervention in ESI+ mode. (**A**) OPLS-DA score plots between HF10L and HF groups. (**B**) OPLS-DA score plots between HF10H and HF groups. (**C**) Permutation test of OPLS-DA model between HF10L and HF groups. (**D**) Permutation test of OPLS-DA model between HF10H and HF groups.

After 8 weeks of intervention, UHPLC-Q-TOF/MS was used to analyze the liver samples from the obese control group and FRT10 treatment group. The metabolite profiles of the liver were altered in the HF group compared to the normal-chow group in our previous study [21], while the metabolites after FRT10 intervention were distinct from the HF group as observed by OPLA-DA score plots for the positive mode (Figure 1). The parameters of OPLS-DA model showed that the model has an excellent modeling and predictive ability, with R^2^X = 0.797, R^2^Y = 0.994, and Q^2^ = 0.507 for HF10L vs. HF, and R^2^X = 0.776, R^2^Y = 0.994, and Q^2^ = 0.542 for HF10H vs. HF. The results showed that the model has no evidence of over-fitting and has high general applicability.

### 3.2. Effect of L. Plantarum FRT10 on Liver Tissue Metabolomics

To determine the discriminating effect of variables between the two comparison groups, variables with VIP > 1 and *p* < 0.05 was further analyzed by Student’s *t*-test of OPLS-DA modeling. In total, 26 metabolites were preliminarily identified as potential biomarkers for the anti-obesity effect of FRT10 analyzed in ESI+ and ESI− mode (Table 1). The potential biomarkers related to glycerophospholipid metabolism included: choline, glycerophosphocholine, CDP-choline, phosphorylcholine, and sn-glycerol 3-phosphoethanolamine. Compared to the HF group, FRT10 administration led to a significant decrease in choline and phosphocholine (*p* < 0.05) and marked decrease in glycerophosphocholine. Taurocholate and cholic acid involved in primary bile acid biosynthesis in HF10L and HF10H groups were reduced 0.30 and 0.45-fold, respectively. After FRT10 intervention, eight amino acids related to amino acid metabolism, such as l-phenylalanine, tyramine, l-threonine, d-ornithine, l-asparagine, d-proline, and l-pyrogutamic acid, were significantly enhanced, while L-histidine was markedly increased. Additionally, 10 potential biomarkers linked to pyrimidine and purine metabolism, including thymidine, uracil, allantoin, inosine, xanthine, xanthosine, and deoxyinosine were significantly increased 1.23 to 4.90-fold (*p* < 0.05), while uridine was significantly decreased 0.55-fold (*p* < 0.05) in the HF10L group as compared to the HF group. Moreover, FRT10 intervention markedly increased thymine and markedly decreased hypoxanthine in HFD-fed obese mice.

### 3.3. Multivariate Statistical Analysis of Cecal Contents

**Figure 2:**
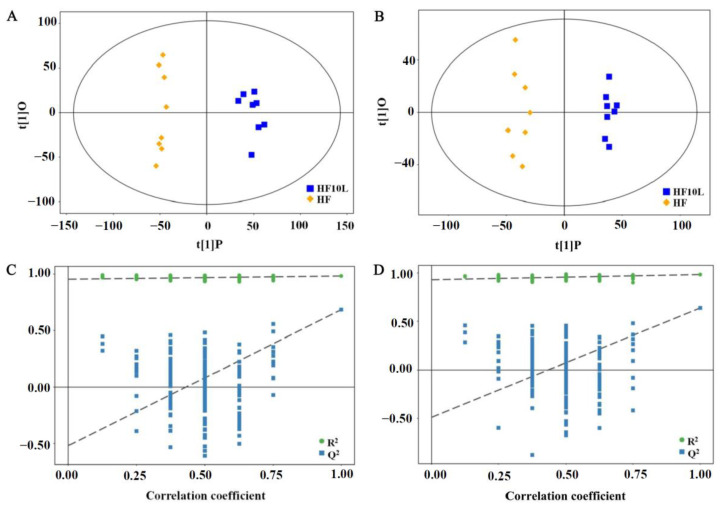
Score plots and permutation test of cecum contents after *L. plantarum* FRT10 intervention in ESI+ and ESI− mode. (**A**) OPLS-DA score plots between HF10L and HF groups. (**B**) OPLS-DA score plots between HF10H and HF groups. (**C**) Permutation test of OPLS-DA model between HF10L and HF groups. (**D**) Permutation test of OPLS-DA model between HF10H and HF groups.

Through the gut–liver axis, intestinal microbial metabolites reach the liver and affect liver metabolism. The profiles of metabolites identified in the cecal contents were altered in HF group compared to the normal-chow group in our previous study [21], while the metabolites after FRT10 intervention were distinct from the HF group as observed by OPLA-DA score plots for both the positive and negative-ion modes (Figure 2). The parameters of OPLS-DA model were as follows: R^2^X = 0.246, R^2^Y = 0.983, and Q^2^ = 0.684 in ESI+ mode, R^2^X = 0.248, R^2^Y = 0.984, and Q^2^ = 0.638 in ESI− mode, which indicated the goodness of fit of the data.

### 3.4. Effect of L. plantarum FRT10 on Cecal Contents Metabolomics

The candidate metabolites were selected as potential biomarkers according to VIP > 1 and *p* < 0.05 of OPLA-DA models. According to the above criteria, a total of 15 endogenous metabolites were screened from cecum contents closely involved in the effect of FRT10 feeding on HFD-fed mice by UHPLC-Q-TOF/MS (Table 2). Six metabolites were increased, and nine decreased in FRT10-treated mice compared with HFD-fed mice. The levels of PC(O-16:0/2:0) and PC(0:0/20:4(5Z,8Z,11Z,14Z)) were significantly decreased 0.65 and 0.02-fold, while PC(O-8:0/O-8:0) and PC(O-14:1(1E)/0:0) were significantly enhanced 1.94 and 1.64-fold. The levels of LysoPC(18:1(9Z)), LysoPC(20:3(5Z,8Z,11Z)), LysoPE(0:0/20:0), PS(18:2(9Z,12Z))/22:6(4Z,7Z,10Z,13Z,16Z,19Z), and 1-Palmitoyl-2-linoleoyl PE were significantly reduced, while LysoPE(0:0/20:4(5Z,8Z,11Z,14Z)) and PA(18:0/18:1(9Z)) were significantly increased. Additionally, 2-amino-3-(3,4-dihydroxyphenyl)propanoic acid and taurocholic acid were reduced, while cholacalcioic acid and xanthine were significantly enhanced. Glycerophospholipid metabolism, primary bile acid biosynthesis, and purine metabolism were the metabolic pathways in which these metabolites were mainly involved.

## 4. Discussion

**Figure 3:**
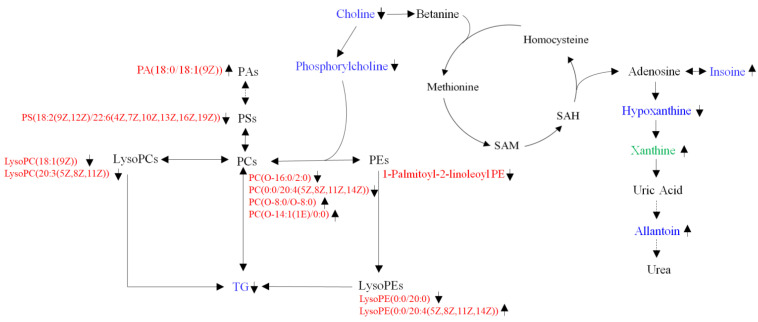
Schematic diagram of the metabolic pathway of FRT10 intervention in obesity according to metabolites analysis of liver and cecum contents. Blue indicated the presence of metabolites in the liver, red indicated the presence of metabolites in cecum contents, and green indicated the presence of metabolites in both liver and cecum contents. ↑ indicated an upward trend and ↓ indicated a downward trend. DAG—diglyceride; TG—triacylglycerols; SAM—S-adenosylmethionine; SAH—S-adenosine homocysteine; PC—phosphatidylcholine; PA—phosphatidic acid; PS—phosphatidylserine; PE—phosphatidylethanolamine.

The gut microbiota produces a variety of compounds that play an important role in modulating distal organ activity, and the liver is located downstream of the intestine, indicating the significance of the gut–liver axis [23]. As a promising anti-obesity candidate, microbiota regulation and its anti-obesity effects on *L. plantarum* have been elucidated by different researchers [16,24,25]. In our previous study, *L. plantarum* FRT10 intervention reduced the extent and features of obesity in HFD-induced obese mice by modulating the structure of gut microbiota [11]. Meanwhile, metabonomics studies of liver and cecum contents showed that after 8 weeks of continuous administration of HFD, the obese model of mice was successfully established and developed a metabolic disorder [21]. In this study, UHPLC-Q-TOF MS was used to detect liver, and cecum contents metabolites of HFD-induced obese mice interfered with FRT10, and multivariate statistical analysis comparisons were made. We found that the intake of FRT10 altered the metabolic profiles of liver and cecum contents, and these changes were partially associated with lipid metabolism and obesity-related diseases. The results contributed to understanding the mechanism by which *L. plantarum* alleviated obesity. Since a lower dose of FRT10 had a better effect on alleviating obesity [11], we focus on the HF10L group in the following study. As shown by our study results, 26 identified liver metabolites and 15 cecum contents were selected as potential biomarkers for FRT10 anti-obesity. Based on identified metabolites, the metabolic pathways of FRT10 intervention against obesity and the analysis of the gut–liver axis are shown in Figure 3.

Dysmetabolism of glycerolphospholipid and fatty acid has been found to be directly associated with the progression of hyperlipidemia. Choline metabolism is disrupted in the context of dysbiosis, and choline deficiency is related to obesity. Choline, synthesized primarily by intestinal biota and completely metabolized in the liver, is an essential nutrient for maintaining the liver function, like phospholipids [26,27]. As shown in Table 1, compared to the HF group, choline, and phosphorylcholine in hepatic metabolites were significantly decreased after FRT10 intervention. Our previous study showed that excess choline and glycerolphospholipid metabolites were positively correlated with obesity [21]. Increased choline in the liver is related to an increased risk of hepatic steatosis, NASH, and lobular inflammation [28]. The result indicated that FRT10 intervention promotes choline metabolism, which is in agreement with the fact that reduced choline bioavailability may lead to NAFLD [29,30]. Choline is an important precursor of phosphatidylcholine (PC), which is mainly the composition of phospholipid-coated very-low-density lipoprotein (VLDL) particles. Glycerophospholipids are the most abundant lipids and the main components of bile and membrane surfactants except biofilms, which are classified into PC, phosphatidylglycerol (PG), phosphatidylinositol (PI), phosphatidic acid (PA), phosphatidylethanolamine (PE), and phosphatidylserine (PS), as well as lyso-PL hydrolyzed according to the fatty acid side chain with different polar heads. In cecum metabolites, PC(O-16:0/2:0) and PC(0:0/20:4(5Z,8Z,11Z,14Z)) were significantly down-regulated, and PC(O-8:0/O-8:0) and PC(O-14:1(1E)/0:0) were significantly up-regulated. PC(O-16:0/2:0) is a neurotoxic lipid species enhanced in Alzheimer’s Disease, which is associated with promoting mitochondrial dysfunction [31]. Altered levels of lysophosphatidylcholines (LysoPCs) have been found to be related to abnormal energy states, such as hyperlipidemia and obesity [32]. LysoPCs are usually produced by hydrolysis of oxidized PC. LysoPC(18:1(9Z)) and LysoPC(20:3(5Z,8Z,11Z)) were significantly reduced in mice fed FRT10. LysoPCs can cause cyclooxygenase expression, inflammation, and autoimmune response, either alone or by activating specific G-protein-coupled receptors [33]. Studies have linked LysoPCs to a number of diseases, including atherosclerosis, sepsis, diabetes, and cancer [34]. LysoPE(0:0/20:0) was significantly decreased, and LysoPE(0:0/20:4(5Z,8Z,11Z,14Z)) was significantly increased after FRT10 intervention, which was partly matched with our previous study that LysoPE(18:1(9Z)) and LysoPE(16:1(9Z)0:0) were positively correlated with obesity, while LysoPE(0:0/20:3(11Z,14Z,17Z)) and LysoPE(20:1(11Z/0:0)) (20:2) were negatively associated with obesity [21]. In addition to PC species, PS(18:2(9Z,12Z))/22:6(4Z,7Z,10Z,13Z,16Z,19Z) and 1-Palmitoyl-2-linoleoyl PE were significantly reduced after FRT10 intervention, which is associated with excess PS in obese mice caused by HFD in our previous study [21]. PA is a common phospholipid that is a synthetic precursor for other lipids. It can be synthesized from and converted into a large number of glycerophospholipids, which are involved in membrane formation, cell signaling transduction, lipid storage, etc. [35]. Interestingly, PA(18:0/18:1(9Z)) was increased 9.78-fold after FRT10 intervention. The result can be interpreted as an attempt by the gut to maintain phospholipid balance by enhancing the PA level. The results suggest that different glycerophospholipids may have different functions, which need to be further studied and clarified. These metabolic alterations of glycerophospholipid metabolites help us further understand the underlying mechanism of FRT10 against obesity.

Bile acids (BA) are important amphipathic signaling molecules and have important physiological functions, and their homeostasis is regulated by gut microbiota. Disturbed bile acids homeostasis may be caused by impaired liver uptake and elevated basolateral bile acids efflusion [36]. After FRT10 intervention, the major bile acid cholic acid (CA) in the liver was significantly decreased by 0.45-fold, while CA was significantly increased by 1.71-fold in the cecum. Free BA solubilizes intestinal lipids with low reabsorption efficiency, resulting in increased BA excretion in feces and replacement of free BA by bile acids newly synthesized from cholesterol in the liver as a mechanism by which excess cholesterol is excreted through the gut–liver axis. The content of taurocholic acid (TCA) in the liver and cecum decreased by 0.30 times and 0.42 times, respectively. TCA is a primary conjugated bile acid synthesized from CA. The result is consistent with a study of higher serum total and conjugated bile acids in patients with liver damage [37]. Our result is consistent with the report that *L. plantarum* H-87 prevented HFD-induced obese mice by regulating bile acid enterohepatic circulation [38].

Amino acids are not only essential nutrients and the source of energy for the human body but also participate in many biochemical processes such as the biosynthesis of purines and the production of uric acid [39]. The primary organ for amino acid metabolism is the liver, which plays an important role in maintaining amino acid homeostasis. Abnormal alterations in amino acids have been found to result in dysregulation that may potentially affect fatty acid, protein, and urea synthesis, energy metabolism, proteolysis, and cell signaling [40]. Eight hepatic metabolites related to amino acid metabolism, including l-phenylalanine, tyramine, l-threonine, d-ornithine, l-asparagine, d-proline, and l-pyrogutamic acid, were significantly enhanced (*p* < 0.05), while l-histidine was markedly increased after FRT10 intervention. These altered amino acids included l-phenylalanine, l-asparagine, and d-proline, which are commonly used as substrates for hepatic glucogenesis [41,42], and the decrease in hepatic amino acid levels caused by long-term HFD may be related to HFD-induced hepatic abnormal gluconeogenesis [43]. The results of the overall enhancement of amino acids partially matched those of a previous study that *Platycodon grandiflorum* ameliorated obesity through significantly increasing serine, glycine, threonine, glutamate, methionine, ornithine, phenylalanine, tyrosine, and lysine in the liver of obese mice [44]. The supplementation of *Luffa cylindrica* could significantly alleviate hepatic steatosis in HFD-induced obese mice by inhibiting lipid synthesis and increasing liver levels of amino acid [45]. Moreover, phenyllactic acid (PLA), a product of phenylalanine catabolism, was significantly increased in the liver. Manna et al. reported that alcohol-treated Ppara-null mice had elevated levels of PLA in urine [46,47]. PLA is a pharmaceutical component used in the synthesis of the hypoglycemic agent englitazone, a non-protein amino acid statin, and a novel intestinal anthelmintic agent PF1022A [48,49]. PLA was reported to alleviate *Samonella Typhimurium*-induced colitis [50]. Therefore, it is necessary to further study the relationship between the change in PLA level and anti-obesity effects.

We also found some changes in metabolites associated with purine and pyrimidine metabolism. Hepatic metabolites such as inosine, xanthosine, xanthine, deoxyinosine, uracil, allantoin (5-ureidohydantoin or 5-ureidoacetolactam), thymidine, and thymin were significantly increased, while hypoxanthine and urdine were markedly decreased. The data were partially concordant with a previous study that HFD induced a significant decrease in hepatic uracil, uridine, inosine, and hypoxanthine, while *Platycodon grandiflorum* treatment alleviated HFD-induced obesity by significantly enhancing the contents of uracil and hypoxanthine [44]. Inosine is usually converted from purine nucleoside phosphorylase to hypoxanthine, which is then converted from xanthine oxidase to xanthine. Xanthine is a host–microbial co-metabolite widely present in body tissues, body fluids, and gut microbiota, which can be converted to uric acid in mice. Both xanthine and hypoxanthine are oxypurines and precursors of uric acid, which are oxidized by xanthine oxidoreductase in purine catabolism [51]. Hypoxanthine, an upstream metabolite of xanthine, was markedly decreased in the liver after FRT10 intervention, which is consistent with significantly higher plasma hypoxanthine levels in obese participants than in lean participants [52]. The hypoxanthine level in plasma was positively correlated with BMI [53]. However, the result contradicted that topiroxostat suppressed body weight gain by increasing hepatic hypoxanthine in diabetic obese mice [54]. Xanthine was found to be significantly increased both in liver and cecum contents. Gut microbiota mediated-xanthine metabolism correlates with HFD-induced obesity [55]. The results are not in accordance with the report of elevated urine xanthine levels in obese mice [55]. Uridine is a pyrimidine nucleoside that plays an important role in regulating glucose and lipid metabolism. Hepatic uridine was significantly decreased, which contradicted that uridine attenuates obesity and ameliorates hepatic lipid accumulation in HFD-fed mice [56]. Uracil was significantly enhanced, which is in agreement with the fact that uracil and ribose-containing uridine have protective effects on drug-induced hepatotoxicity and mental disorders [57]. Allantoin is a diureide of glyoxylic acid, which has been reported to have antidiabetic effects [58,59], which was also found to be significantly increased in liver after FRT10 intervention. From these results, it appears that FRT10 attenuated obesity by enhancing purine and pyrimidine metabolism, resulting in the predisposition of the body toward catabolism in obese mice. These findings suggest that gut microbial-mediated xanthine metabolism is involved in resistance to HFD-induced obesity.

## 5. Conclusions

This study verified the anti-obesity effect of FRT10 by metabonomics. Liver and cecum metabonomic studies based on UPLC-Q-TOF/MS revealed the modulation effects of FRT10 on obese mice. Based on identified metabolites, we found that FRT10 alleviated obesity via metabolic pathways in glycerophospholipid metabolism, primary bile acid biosynthesis, amino metabolism, and purine and pyrimidine metabolism. FRT10 appeared to be a promising candidate for probiotics to combat the increasing incidence of metabolic syndrome and obesity.

## Figures and Tables

**Table 1:** Metabolites alterations in liver between FRT10 treatment group and obese control group.

Metabolites	RT (s)	*m*/*z*	Fold Change	
			HF10L vs. HF	HF10H vs. HF
Choline	780.035	104.1058352	0.83 *	1.55 *
Glycerophosphocholine	862.561	258.1094388	0.61 +	0.51 +
CDP-choline	878.204	489.1135327	-	0.52 +
Phosphorylcholine	717.05	242.0794821	0.28 *	0.63 *
sn-Glycerol 3-phosphoethanolamine	788.63	198.0517674	-	0.67 *
Taurocholic acid	413.829	498.2875244	0.30 *	-
Cholic acid	391.737	373.2730818	0.45 *	-
Phenyllactic acid	521.458	149.0586964	1.18 *	1.18 *
L-Phenylalanine	542.1065	166.0855048	1.29 *	1.27 *
Tyramine	521.673	120.0799482	1.21 *	1.21 *
L-Threonine	704.9	120.0647667	1.23 *	1.21 *
D-Ornithine	1012.455	115.0853902	1.18 *	1.24 *
L-Asparagine	750.138	133.0599344	1.16 *	1.12 +
D-Proline	1012.13	116.0694156	1.17 *	1.27 *
L-Pyroglutamic acid	745.28	147.0754241	1.33 *	-
L-Histidine	1015.6	156.0760477	1.22 +	1.25 +
Thymine	283.614	127.0491779	1.45 +	-
Thymidine	180.4075	241.0827884	3.33 *	2.66 *
Uracil	149.349	111.0197557	1.56 *	1.65 *
Allantoin	367.132	159.0505486	1.23 *	-
Uridine	321.659	245.0764623	0.55 *	0.45 *
Inosine	236.175	249.0626869	1.63 *	1.86 *
Hypoxanthine	412.355	137.0448623	0.16 +	0.53 +
Xanthine	428.656	151.0258253	4.90 *	2.24 +
Xanthosine	394.365	283.067998	2.24 *	2.38 *
Deoxyinosine	325.282	251.078228	2.94 *	-

The ratio between HF10L vs. HF or HF10H vs. HF is shown in the table as fold change. “*” indicates significant differences in metabolites between the two groups compared with VIP > 1 and *p* < 0.05. “+” indicates marked differences in metabolites between the two groups compared with VIP > 1 and 0.05 < *p* < 0.1. RT retention time.

**Table 2:** Metabolites alterations in cecum contents between HF10L and HF groups.

Metabolites	RT (s)	*m*/*z*	Fold Change HF10L vs. HF
PC(O-16:0/2:0)	330.811	524.3154739	0.65
PC(0:0/20:4(5Z,8Z,11Z,14Z))	401.017	543.3885338	0.02
PC(O-8:0/O-8:0)	469.048	482.3602632	1.94
PC(O-14:1(1E)/0:0)	286.959	451.3413931	1.64
LysoPC(18:1(9Z))	458.095	478.2949417	0.14
LysoPC(20:3(5Z,8Z,11Z))	458.234	545.4042362	0.14
LysoPE(0:0/20:0)	477.56	510.3550402	0.14
LysoPE(0:0/20:4(5Z,8Z,11Z,14Z))	392.452	501.3202624	2.69
PS(18:2(9Z,12Z))/22:6(4Z,7Z,10Z,13Z,16Z,19Z)	508.345	832.5033673	0.14
PA(18:0/18:1(9Z))	558.79	700.557389	9.78
1-Palmitoyl-2-linoleoyl PE	540.667	715.4910486	0.68
2-amino-3-(3,4-dihydroxyphenyl)propanoic acid	216.208	197.1284116	0.61
Cholic acid	392.413	373.2732771	1.70
Taurocholic acid	302.382	514.2852392	0.42
Xanthine	62.5003	152.0540194	1.83

PC—phosphatidylcholine; PA—phosphatidic acid; PE—phosphatidylethanolamine; PS—phosphatidylserine; LysoPCs—lysophosphatidylcholines; LysoPE—lysophosphoethanolamine; RT—retention time.

## Data Availability

Data is contained within the article.
